# Predictive factors for moderate or severe exacerbations in asthma patients receiving outpatient care

**DOI:** 10.1186/s12890-017-0422-6

**Published:** 2017-05-02

**Authors:** Francisco Javier Álvarez Gutiérrez, Marta Ferrer Galván, Juan Francisco Medina Gallardo, Marta Barrera Mancera, Beatriz Romero Romero, Auxiliadora Romero Falcón

**Affiliations:** Asthma Unit, UMQER, Hospital Universitario Virgen del Rocio, C/Alcalde Manuel del Valle, edificio Cónsul, Portal 6, 1° A, 41008 Sevilla, Spain

**Keywords:** Asthma management, Asthma control test, Atopic, Functional and inflammatory parameters, Disease control, Predictive factors for exacerbations

## Abstract

**Background:**

Asthma exacerbations are important events that affect disease control, but predictive factors for severe or moderate exacerbations are not known. The objective was to study the predictive factors for moderate (ME) and severe (SE) exacerbations in asthma patients receiving outpatient care.

**Methods:**

Patients aged > 12 years with asthma were included in the study and followed-up at 4-monthly intervals over a 12-month period. Clinical (severity, level of control, asthma control test [ACT]), atopic, functional, inflammatory, SE and ME parameters were recorded. Univariate analysis was used to compare data from patients presenting at least 1 SE or ME during the follow-up period vs no exacerbations. Statistically significant (*p* <0.1) factors were then subjected to multiple analysis by binary logistic regression.

**Results:**

A total of 330 patients completed the study, most of whom were atopic (76%), women (nearly 70%), with moderate and mild persistent asthma (>80%). Twenty-seven patients (8%) had a SE and 183 had a ME (58.5%) during follow-up. In the case of SEs, the only predictive factor identified in the multiple analysis was previous SE (baseline visit OR 4.218 95% CI 1.53-11.58, 4-month follow-up OR 6.88 95% CI 2.018-23.51) and inhalation technique (OR 3.572 95% CI 1.324-9.638). In the case of MEs, the only predictive factor found in the multiple analysis were previous ME (baseline visit OR 2.90 95% CI 1.54-5.48, 4-month follow- up OR 1.702 95% CI 1.146-2.529).

**Conclusions:**

The primary predictive factor for SE or ME is prior SE or ME, respectively. SEs seem to constitute a specific patient "phenotype", in which the sole predictive factor is prior SEs.

## Background

Asthma exacerbations affect the clinical course of the asthma patient. Although different studies have used different definitions of asthma exacerbation, it is generally accepted that severe exacerbations (SE) are those requiring urgent intervention, defined as prescription of systemic steroids (oral or injected) or dose increase of maintenance steroids for at least 3 days, visit to the emergency department or hospitalisation due to aggravation of symptoms. Moderate exacerbations (ME) have been defined as any deterioration in the patient's symptoms or lung function, beyond day-to-day variations associated with the disease requiring a change of medication but that do not meet severe criteria [1]. Treatment is escalated to prevent the exacerbation from becoming severe. Exacerbations are a fundamental component of the current approach to future risk. Risk factors must be reduced for effective control of the disease [2].

Several studies have attempted to determine which factors can predict SEs, and an evaluation of these elements can contribute towards reducing the impact of exacerbations and confirm the existence of a group to be “at risk” patients that merit special monitoring. Exacerbation predictors suggested to date include factors such as current control (based on the GINA severity classification), or clinical control tests [3–7]. Other parameters put forward as predictors for exacerbation are social factors, the healthcare system itself [8], less use of inhaled steroids [9], other comorbidities such as rhinitis [10], or recent SE [6, 10–12]. One of the factors described (poorly controlled disease) is still one of the most pressing problems associated with the treatment of asthma. Recent studies have found a high percentage of patients with poorly controlled disease [13, 14], even though modern asthma therapy is known to be effective in most patients.

Although a large number of studies in predictive factors for SEs in asthma have been published, most of these include patients seen in emergency departments or presenting with serious or "near fatal" asthma. Few studies have focussed on evaluating the predictive factors for exacerbation in patients receiving outpatient care, and even fewer have attempted to identify factors capable of predicting SEs, and a possible relationship between both types of exacerbation has not been confirmed.

The aim of our study was to evaluate predictive factors for exacerbations in a large group of patients followed up for 12 months in an outpatient asthma clinic. To our knowledge, this is the first study in terms of setting (pulmonology outpatient clinics), type of patient (mostly mild to moderate), and type of exacerbation (both moderate and severe).

## Methods

Prospective study conducted from March 2007 to March 2010 in asthma outpatient clinics attached to a Pulmonology Department.

### Patients

Inclusion criteria were: patients aged over 12 years, previously diagnosed with asthma according to GINA 2006 [15] clinical and functional criteria that had not received oral steroids during the month prior to their inclusion; current or former smokers, with accumulated consumption of less than 10 pack years; patients receiving 200 to 2000 mcg/day fluticasone or equivalent dose of budesonide, alone or associated with long-acting beta-2 agonist, with/without 10 mg montelukast every 24 h. Exclusion criteria were: Patients with history of other respiratory diseases (COPD, bronchiectasis, interstitial or tumour diseases, etc.); patients with severe asthma treated with long-term oral steroids; exacerbation at the time of recruitment; administration of systemic steroids in the past month.

### Methodology

On the day each patient was recruited for the study, the researcher, following protocol, noted their epidemiological and clinical variables. Once they had been examined by the attending doctor, the patient was handed over to a registered nurse for ACT, FeNO, forced spirometry, and allergy testing, if this had not been performed previously. Patients with long-term treatment were told to suspend the last dose prior to undergoing functional tests. FeNO was measured by means of an electrochemical device (NIOX MINO* aerocrine, Solna Sweden). Forced spirometry was measured using Master Scope PC Viasys Healthcare spirometers and JLab, Lab Manager, V 5.3.0, software, following ATS/ERS [16] recommendations. A baseline test followed by a further test after administration of 200 mcg of salbutamol (postbronchodilator) was performed. FEV1, FEV1/FVC ratio, and FEV1 % change from baseline and postbronchodilator values were expressed as absolute values and % of predicted value.

Patients completed the ACT (Asthma Control Test) questionnaire, comprising 5 questions related to asthma symptoms and use of asthma medication during the 4 previous weeks [17]. The sensitization test was carried out with the standard allergen prick test used in our hospital [18]. Atopy was defined on the basis of a positive prick test.

Based on the results of the patient-doctor interview and the functional tests, patients were assigned a level of severity (intermittent, persistent mild, moderate or severe) and the level of control was established according to GINA 2006 [15] (controlled, partially controlled, or uncontrolled).

SE (severe exacerbation): was defined as an exacerbation meeting at least 1 of the following conditions: a) use of systemic steroids, or increased maintenance dose - in this case, a course of oral steroids or an increase in the maintenance dose lasting from 3 to 30 days, based on symptom severity and the opinion of the attending pulmonologist; b) hospitalisation or treatment in the emergency department due to the need for systemic steroids to control the exacerbation.

ME (moderate exacerbation), was defined as: aggravation of the patient's usual symptoms requiring increased rescue bronchodilator use at least for 2 days so the patient need finally to increase maintenance medication but no oral o sistemic steroids, no hospitalisation and no treatment in the emergency departement.

Uncontrolled days were defined as the number of days during which the patient had to use rescue medication or increase their maintenance dose to control their asthma symptoms.

Unscheduled medical care was defined as a spontaneous medical consultation requested by the patient due to symptoms they were unable to control by themselves.

A complete blood count, including eosinophils, was ordered at the baseline visit.

Patients were given a symptom diary and asked to note down all clinical events over the study period. The diary was checked at 4, 8 and 12 months of follow-up. During these follow-up visits, patients were evaluated according to their GINA level of control and ACT. Spirometry (reversibility testing) and FeNO testing were performed, and the patient was asked about the study variables (SE, ME, unscheduled medical care, uncontrolled days, treatment given). Treatment compliance was evaluated by the attending nurse on the basis of the patient's own assessment, and classified as good (more than 75% of the prescribed dose), fair (50% - 75% of dose), or poor (<50% of dose). Inhaler technique was also checked during the interview, and classified as correct or incorrect. At the final visit, a new complete blood count, including eosinophils, was ordered.

All patients were asked to sign an informed consent form authorising inclusion of their clinical data in the study database. At no time were the patient's personal details entered into the database; instead, they were assigned a reference number that correlated with their medical records, which were stored in separate files under the custody of the researchers. This study was approved by the hospital's independent ethics committee.

### Statistical analysis

Data were analysed with the statistical analysis programme SPSS v. 17. Qualitative variables were expressed as percentages, and quantitative variables as mean plus standard deviation (SD), minimum, maximum and percentiles 25,50 and 75.

To analyse differences between patients presenting one or more SEs and MEs and the rest of patients, the independent samples *t* test was used for quantitative variables, while the related sample *t* test was used to analyse differences in follow-up variables. Prior to this, the groups were tested for homogeneity of variance (Levene's test). The Mann Whitney *U* test or the Wilcoxon matched pairs was used to compare ordinal independent variables. The Chi-square test was used to evaluate differences between qualitative variables.

All variables with a statistical significance of < 0.1 in the univariate analysis were included in the multivariate binary logistic regression model and the corresponding odds ratios (OR) were assigned to a 95% confidence interval.

Statistical significance was set at *p* <0.05.

## Results

**Fig. 1:**
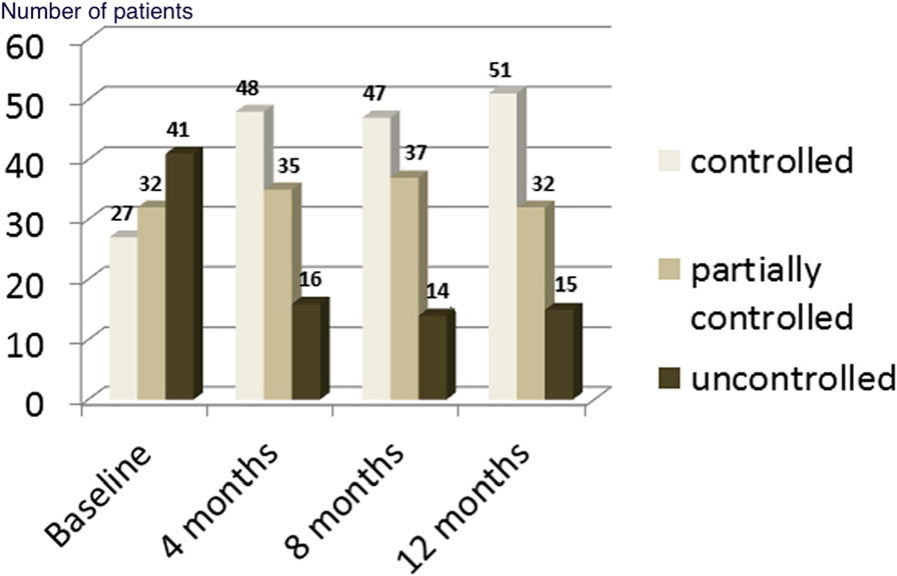
Clinical course of disease control in subsequent follow-ups

A total of 407 patients were included in the study, of which 330 completed all follow-up stages (4, 8 and 12 months), and were included in the final analysis. Mean age was 39.2 (16.7) years, with women being in the majority (69.7%). Table 1 shows the general characteristics of the study patients. Notably, most (more than 80%) patients presented mild or moderate persistent asthma, and 73% presented poorly controlled asthma at the baseline visit (partially controlled or uncontrolled). Table 2 shows the treatment regimens followed by patients at the time of inclusion in the study. More than 23% of patients did not routinely take any kind of controller treatment, and most patients were taking combined therapy (LABA+ inhaled corticosteroid). The table also shows the percentage of therapeutic compliance and inhaler technique. It can be seen that compliance with both oral and inhaled medication gradually improved over the follow-up period. This was also true of inhaler technique, which was considered correct in 96% of patients at the final follow-up. Table 3 shows the clinical course of episodes of SE and ME, unscheduled medical care, and uncontrolled days. Significant differences were observed between the baseline visit and subsequent follow-ups, with significant improvement in all parameters. Figure 1 shows the clinical course of disease control in subsequent follow-ups. It is interesting to note that at the baseline visit, only 27% of patients had controlled disease. This improved to 51% by the final follow-up at 12 months.

**Table 1:** General patient characteristics

*N* = 330	Media	S.D.	Percentiles		
			25	50	75
Age	39.2	16.7	25	37	50.75
BMI	26.96	5.28	23.25	26.31	29.84
FEV1%	96.2	20.4	84	98	110
FEV1:FVC	73	10.9	65.89	74.49	80.68
FEV1 Reversibility	9.32	13	2	6	14
ACT	18	4.7	15	18	22
FENO	37	32.3	15	25	46
Sex	F: 30.3% M: 69.7%				
Smoker	S: 10.9%, FS: 23.6%, PS: 3.3%, NS: 62.1%				
Atopy	Yes: 76.8%				
Severity	Intermittent: 11.8% Mild Persistent: 33% Moderate Persistent: 47.6% Serious Persistent: 7.6%				
Asthma control	Controlled 27% Partially controlled 32.1% Uncontrolled 40.9%				

*ACT* asthma control test. *F* female. *FENO* fracction exhaled nitric oxide. *FEV1* forced expiratory volume in 1 s. *FS* former smoker. *FVC* forced vital capacity. *M* male. *NS* never smoker. *PS* passive smoker. *S* smoker

**Table 2:** Treatment regimen at baseline and follow-up, level of compliance and inhaler technique

*N* = 330	Baseline	4 months	8 months	12 months
No continuing tx (on demand)	23.3%	9.8%	12.6%	9.9%
Inhaled steroids mono-tx	8.9%	8.3%	7.7%	7.1%
Formoterol/budesonide	20.8	26.5%	26.2%	26.2%
Salmeterol/fluticasone	46.9%	52.1%	53.5%	56.5%
Montelukast	32.4%	40.1%	47.9%	50%
Equivalent dose of inhaled steroids	1185.5 (780.1)	1276.6 (922.3)	1249.3 (851.9)	1221.5 (822.9)
Compliance inhaled medication				
Good	74.1%	77.1%	85.8%	86.3%
Fair	5.6%	7.4%	5.1%	6.2%
Poor	20.3%	15.5%	9.2%	7.5%
Compliance oral medication				
Good	72%	81.3%	86.7%	91.1%
Fair	4.8%	2.7%	2.8%	2.6%
Poor	23.2%	15.9%	10.5%	6.3%
Inhaler technique				
Correct	74.1%	88.7%	92%	96.1%

**Table 3:** Evolution of instability parameters over follow-up

*N* = 330	Baseline (prior) X (SD)	4 months X (SD)	8 months X (SD)	12 months X (SD)
Numbers of Moderate exacerbations	0.64(0.96)^a^	0.39(0.88) ^a^	0.39(0.70) ^a^	0.33(0.68) ^a^
Numbers of Serious exacerbations	0.07(0.29) ^b^	0.04(0.22)	0.03(0.21)^b^	0.03(0.18)^b^
Numbers of unscheduled consultations	0.58(1.07) ^a^	0.25(0.74) ^a^	0.34(1.08) ^a^	0.23(0.58) ^a^
Numbers of uncontrolled days	66.8(45.77) ^a^	46.9(44.05) ^a^	44.5(43.5) ^a^	41.2(42.9) ^a^

^a^ *p* <0.001 (paired *t* test) Baseline and 4–8- and 12 months follow-ups

^b^ *p* <0.1 (paired *t* test) Differences at baseline and 8- and 12-months follow-up

(Significant improvement in all parameters were observed between baseline visit and subsequent follow-ups)

### Serious exacerbations

Twenty seven patients presented an SE during the follow-up period (8.2% of the total cohort). When we evaluated the differences between patients presenting an SE during follow-up vs those that did not on the basis of their baseline general and clinical characteristics, pulmonary function and FeNO, the univariate analysis showed significant differences in the equivalent dose of inhaled steroids (*p* <0.01), inhaler technique (*p* <0.03), number of serious exacerbations in the previous 4 months (*p* <0.001) and severity of disease (*p* <0.01).

Using these same variables to analyse 4-month follow-up data from patients with an SE at between 4 and 12 months, we also found significant differences in the number of previous SEs, (0-4 months) (*p* <0.01).

Statistically significant variables at the baseline interview were included in the multivariate analysis. This showed that only previous SEs correlated with SEs during the following year (OR 4.218 [95% CI 1.536-11.588]) and inhalation technique (OR 3.572 [95% CI 1.324-9.638]). When we included statistically significant variables from the 4-month follow-up, we also found SE in the previous 4 months to be the only associated factor (OR 6.889 [95% CI 2.018-23.512]), (Table 4).

**Table 4:** Multiple (binary logistic regression) analysis of baseline and 4-month followup variables associated with serious and moderate exacerbations in subsequent follow-ups

Analysis of baseline variables associated with SEs the following year			
	OR	CI 95%	p
Prior serious exacerbations(4 months prior to baseline visit)	4.218	1.536–11.588	0.005
Technique	3.572	1.324–9.638	0.012
Analysis of baseline variables associated with MEs the following year			
Prior moderate exacerbations (4 months prior to baseline visit)	2.909	1.542–5.489	0.001
Analysis of 4-month follow-up variables associated with SEs at 4 to 12 months			
	OR	CI 95%	p
Prior serious exacerbations (0–4 months)	6.889	2.018–23.512	0.002
Analysis of 4-month follow-up variables associated with MEs at 4 to 12 months			
Prior moderate exacerbations (0–4 months)	1.702	1.146–2.529	0.008

### Moderate exacerbations

A total of 193 patients (58.5%) presented 1 or more moderate exacerbations over the 12-month follow-up period, univariate analysis of baseline data interview to compare patients presenting an ME and those that did not showed significant differences in the number of previous MEs (*p* <0.001), number of SEs, (*p* <0.04), sex (*p* <0.04), equivalent maintenance dose of inhaled steroids (*p* <0.05), while borderline significant differences (included in the multivariate analysis) were found in BMI (*p* <0.07) and postbronchodilator FEV1 (*p* <0.09). Analysing these same variables for patients with an ME at 4 and 12 months at the 4-month follow-up, we found significant differences in ACT (*p* <0.001), GINA control (*p* <0.001), previous ME episodes (0-4 months) (*p* <0.001), FENO (*p* <0.02), previous uncontrolled days (*p* <0.01), and absolute FEV1 (*p* <0.05).

In the multiple analysis, the number previous MEs correlated with MEs during the following year (OR 2.909 95% CI 1.542-5.489) and MEs in the previous 4 months was t related with MEs (OR 1.702 95% CI 1.146-2.529).

## Discussion

In this study, we prospectively evaluated asthma patients followed up in outpatient pulmonology clinics to determine factors that could predict an SE or ME in the following year. The multiple analysis showed that previous SEs at both the baseline interview and 4-month follow-up is the only predictive factor for SE in the future. The only predictive factor for ME at baseline visit was previous ME, while the 4-month follow-up ACT test score was a predictive factor.

Several different studies have evaluated predictive factors for asthma exacerbations. However, the patient profile in these studies usually differs from ours, which included patients receiving treatment in pulmonology outpatient clinics. Under our inclusion criteria, most patients presented mild or moderate asthma, with a small group of severe asthma patients. Patients with more serious disease and with more previous exacerbations, or those taking oral steroids in the month prior to the baseline interview, were excluded. Our aim in including this type of patient was to study clinical and functional variables and inflammatory parameters without interference from changes in the patient's usual status brought about by intensive recent treatment with oral steroids. In view of the follow-up schedule (4-monthly), we did not include patients whose baseline severity required more frequent follow-up. Because of this, the results of our study cannot, a priori, be generalized to patients with more severe disease. As a result, relatively few of our study subjects presented an SE during follow-up (only 27, or around 8% of the total). Nevertheless, the predictive factor uncovered in the multivariate analysis (previous SE) is consistent with the findings of other studies, such as Miller et al. in 2007 [12], a prospective study of 2,780 patients aged > 12 years in the US diagnosed with severe or difficult-to-treat asthma (TENOR study). In this project, the factor most strongly related with future exacerbations was recent exacerbations (OR 6.33). Other less important predictive factors were severity of disease (severe asthma) and level of control. These data were used in a more recent study [19] that included the same series studied in the TENOR plus a paediatric population (6–11 years). Other studies, such as that conducted by Peters et al. [6] also found that a history of exacerbations can be predictive of future flare-ups in asthmatic adults. Similarly, the retrospective cohort study of UK asthma patients followed-up in primary care centres published by Price et al. [10] found that patients with allergic rhinitis associated with asthma presented more exacerbations (more visits to the doctor and hospitalisation). They also found that patients needing 1 or more courses of oral steroids to treat exacerbations in the preceding year are 3 times more likely to be hospitalised for asthma the following year. In other studies, need for a course of oral steroids has been shown to be a predictor of need for oral steroid in the future [11, 20].

In our series, we also found that SE in the 4 months prior to the baseline interview, and between this interview and the 4-month follow-up, can predict SE in the future (baseline or 4 to 12 months). Indeed, SE between the baseline interview and 4-month follow-up was the factor with the greatest effect size (OR 6.889). This, therefore, appears to be important not only in patients with severe asthma, but also in patients with different levels of disease severity receiving treatment in outpatient clinics. Other predictors of exacerbation described in the literature include poor compliance with inhaled steroid therapy [9], social factors such as poor healthcare [8], FEV1 values in combination with ACT [21], high FeNO levels [22], and even low FeNO levels [23], and, more recently, high blood eosinophil levels (>400/mm) [24]. Other factors mentioned include poor inhaler technique, which has been associated with poorly controlled disease and the need for unscheduled medical consultation [25]. In our study, we found same conclusions, poor inhalation technique is a predictor factor of future SE at baseline visit. Fortunately, as inhaler technique is a solvable factor we found in following visits the control was good as the inhaler technique (that it was taught by our specialist nursing) is correct in most of patients (Table 2).

Other predictors of future exacerbations include the score obtained in some clinical questionnaires. In this respect, some studies have evaluated the benefits of the Asthma Control Questionnaire (ACQ) [5], the Asthma Therapy Assessment Questionnaire (ATAQ) [6] or more recently, the results of the ACT [4, 7, 12], coming up with significant findings. In our study, we found that ACT is not a significant predictive factor for moderate and severe exacerbations at baseline or follow up visits. Searching the literature, we were unable to find any studies exclusively evaluating predictive factors for MEs. Kupcyk et al. performed a multiple analysis of both SE and ME together, finding that FENO levels > 45 ppb and history of smoking were the only factors related with frequent exacerbations (2 or more events per year) [26].

The limitations of our study are mainly due to the type of patient included. The decision to exclude patients with more severe disease or more prior exacerbations probably prevented us from finding other predictive factors. Moreover, prior MEs at the baseline visit were limited to those occurring up to 4 months previously, but excluded the month immediately before starting the study. During this month, according to the protocol, study patients should not have taken systemic steroids. We did not investigate the origin of the exacerbation, and instead relied on the patient's own report. This was because they were mostly treated at the patient's primary healthcare centre or in hospital. We did not use a validated test to determine the level of therapeutic compliance, and instead relied on the patient's own reported compliance. This could underestimate the patient's adherence and the effect of this variable on future exacerbations.

Finally as a limitation of our study is that it has a prospective and unblinded design, that could have influence on the collection of variables and results.

Despite the foregoing, we believe our results can be extrapolated to the study population (patients with mainly mild to moderate disease in specialist. We also believe that these results could reveal a particular SE patient profile or phenotype, irrespective of other clinical, functional or inflammatory parameters.

In conclusion, in our cohort of asthma patients followed-up for 1 year, the main predictive factor for future SE was prior SE, predictive factors for ME were prior ME.

## Conclusions

The primary predictive factor for SE or ME is prior SE or ME, respectively. SEs seem to constitute a specific patient "phenotype", in which the sole predictive factor is prior SEs.

## Abbreviations

ACQ: Asthma control questionnaire
ACT: Asthma control test
ATAQ: Asthma therapy assessment questionnaire
COPD: Chronic obstructive pulmonary disease
FeNO: Fractional exhaled nitric oxide
FEV1: forced expiratory volume in 1 s
FVC: Forced vital capacity
GINA: Global iniciative for asthma
ME: Moderate exacerbations
SE: Severe exacerbations
